# Generative Grammar: A Meaning First Approach

**DOI:** 10.3389/fpsyg.2020.571295

**Published:** 2020-11-23

**Authors:** Uli Sauerland, Artemis Alexiadou

**Affiliations:** ^1^Leibniz-Centre General Linguistics (ZAS), Berlin, Germany; ^2^English Linguistics, Institute of English and American Studies, Humboldt Universität zu Berlin, Berlin, Germany

**Keywords:** generation, cognition, semantics, syntax, morphology, sociolinguistics, bilingualism, scope

## Abstract

The theory of language must predict the possible thought—signal (or meaning—sound or sign) pairings of a language. We argue for a Meaning First architecture of language where a thought structure is generated first. The thought structure is then realized using language to communicate the thought, to memorize it, or perhaps with another purpose. Our view contrasts with the T-model architecture of mainstream generative grammar, according to which distinct phrase-structural representations—Phonetic Form (PF) for articulation, Logical Form (LF) for interpretation—are generated within the grammar. At the same time, our view differs from early transformational grammar and generative semantics: We view the relationship between the thought structure and the corresponding signal as one of compression. We specify a formal sketch of compression as a choice between multiple possible pronounciations balancing the desire to transmit information against the effort of pronounciation. The Meaning First architecture allows a greater degree of independence between thought structures and the linguistic signal. We present three arguments favoring this type of independence. First we argue that scopal properties can be better explained if we only compare thought structures independent of the their realization as a sentence. Secondly, we argue that Meaning First architecture allows contentful late insertion, an idea that has been argued for in Distributed Morphology already, but as we argue is also motivated by the division of the logical and socio-emotive meaning content of language. Finally, we show that only the Meaning First architecture provides a satisfying account of the mixing of multiple languages by multilingual speakers, especially for cases of simultaneous articulation across two modalities in bimodal speakers. Our view of the structure of grammar leads to a reassessment of priorities in linguistic analyses: while current mainstream work is often focused on establishing one-to-one relationships between concepts and morphemes, our view makes it plausible that primitive concepts are frequently marked indirectly or unpronounced entirely. Our view therefore assigns great value to the understanding of logical primitives and of compression.

## 1. Introduction

Several species show evidence of the formation of complex mental representations for aspects of their social and physical environment as well as their planned actions (Bermúdez, 2007; Gallistel, 2011; Andrews and Beck, 2017; Chemla et al., 2019, and others). Humans in addition possess a capacity to communicate complex mental representations with other humans that far exceeds that of all other species in terms of scope, flexibility, and communicative success (Hobaiter and Byrne, 2011; Schlenker et al., 2016; Suzuki et al., 2017, and others): human language. Though the importance of language to our species is evident, the scientific study of human language has proved to be difficult and contentious (Harris, 1995; Graffi, 2001; Thomas, 2020, and others). We argue that one of the reasons for this difficulty is that the primacy of meaning—i.e., the complex mental representations we likely share to a large extent with other species—has not sufficiently been taken into account. Aspects of mental representations shared across species are expected to be present in humans too independently of language, even though humans unlike other species can relate complex linguistic signals to these representations. If structured thought exists independent of language, it calls into question most current theorizing on language. Much current work views structure as either emerging from statistical patterns in language use (Goldberg, 1995; Tomasello, 2003) or as a core property of language itself (Chomsky, 2015), which we discuss in detail later on. But then, could structure also be available without language? Other work allows cognitive structure independent of language, but assumes essentially a one-to-one correspondence between cognitive and linguistic structure (Jackendoff, 2002). But then, how could cognitive structure be present without language being available as well? We propose an alternative that conceives of a new view of grammar that we think does better at explaining the link between cognitive and linguistic structure.

**Figure 1:**
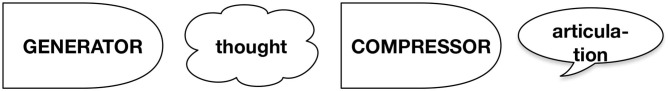
The Meaning First approach to language assumes that structural representations are generated outside of language and then realized by a compressor for communication.

Our proposal for the structure of grammar is sketched in Figure 1, which summarizes our formal sketch in section 2. The term *Thought*-system refers to a cognitive system of humans that is at least partially shared with other species. The *Generator* of the thought-system forms complex thought representations from an inventory of logical primitives and can relate these to memory and to sensory perception of the environment. We view *Language* as a system that relates thought representations to aspects of audiovisual signals in articulation and perception. The Meaning First approach assumes that thought is primary, while language is derived as a realization by the system we call the *Compressor*. Our perspective entails that human thought is organized largely independent of communication, and there is no reason to expect thought representations to be well-suited for communication, while we expect language to be strongly influenced by its communicative function.^1^ Specifically, we argue that language involves a substantial compression of thought representations. This allows us to address evidence for silent structure in language. Consider briefly example (1) which we discuss in more detail in the following section. The present tense auxiliary *do* is optionally pronounced in English with only a slight difference in meaning between the two variants.


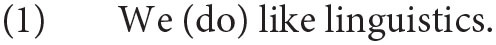


We propose an account in terms of compression that requires speakers to not pronounce “*do”* in normal conditions. If speakers pronounce “*do,”* hearers conclude therefore that conditions are not normal, and a manner implicature is triggered (Grice, 1989). Compression in our view is compatible with non-pronounciation of many parts of a thought structure as long as hearers are sufficiently likely to reconstruct the missing pieces. Compression, as we show in section 2, predicts manner implicatures.

In the following, we first introduce the Meaning First approach in more formal detail focussing on the concepts of *thought* and *compression* in section 2. Then we discuss three key predictions of the Meaning First approach that are listed below in the order we discuss them in this paper. All three arise from the independence of thought and language of the Meaning First approach. Prediction 1 addresses work that argued against *Generative Semantics* almost 50 years ago, which is relevant since the Meaning First approach bears a superficial similarity to Generative Semantics. We show however that the Meaning First approach makes correct predictions concerning the scope properties of sentences and might even compare favorably to other current views. Prediction 2 expands on existing work in Distributed Morphology (Marantz, 1995), in particular its concept of *Late Insertion*. We argue that late insertion within the Meaning First approach can predict key properties of the division of meaning into logical and socio-emotive aspects. Prediction 3 concerns multilingual speakers and is confirmed most directly in bimodal speech where speakers use both modalities simultaneously (Emmorey et al., 2008 and others).

1. The scopal properties of sentences should be determined by their logical properties.
2. Logical and socio-emotive aspects of meaning should systematically differ.
3. Thought may simultaneously access two language systems.

## 2. Thoughts and Compression

The ideas we outlined in the introduction are of a programmatic nature. In this section, we specify the central notions of *thought* and *compression* more formally for concreteness. The formalization is preliminary because doing so requires us to make several specific choices with broad consequences at this point. We hope the formalization can serve as the basis for future empirical work to refine these choices.

One of our basic assumptions is that of a set of primitive concepts *C*. For the following, we can remain agnostic as to how these are to be understood specifically—it could be that primitive concepts are simply markers such as concept-a, concept-b, …(or more evocatively: cause, object, human, …) or it may be a set of mathematical entities as in model-theoretic approaches to semantics. The latter view provides many advantages in our opinion, but this is not the place to argue in favor of it as we can remain agnostic.

One fairly general, formal formulation of *thought* is as follows: let *C* be the set of primitive concepts, and *M*_*C*_ be the set of unordered, binary trees over *C*. *M*_*C*_ is the set of *(possible) concepts*. For our current purposes, we can assume that all possible concepts are actual concepts, but work on concept combinations frequently assumes further well-formedness restrictions, e.g., of a type-theoretic nature (Montague, 1970).^2^

Furthermore, let ⊧ be a partial relation between a possible world *w* and possible concept *c*. We will then say *c* is *true* in *w* iff. *w* ⊧*c*. We say that *c* is a *(propositional) thought* iff. there is a possible world such that *w* ⊧*c*.

Our formulation assumes that concept formation is a binary, commutative recursive operation similar to the operation *Merge* in Chomsky (2015).^3^ Our conception also assumes that possible worlds are sufficient to capture the aspects of human non-conceptual sensory and memory systems relevant to language meaning (see more discussion below). At the same time, the formulation leaves open the recursive specification of ⊧ and a semantic differentiation of concepts (including primitive ones) that are never true of a possible world—i.e., contradictory concepts are not thoughts by the above definition (Chierchia, 2013 and others).

Now we turn to compression. A minimal formal characterization of compression is based on the exponence relation → of Distributed Morphology (Halle and Marantz, 1993; Keine, 2013 and others). Exponence relates some concepts (primitive or complex) to possible *Messages* (or phrases) of the language including a null message ∅. For concreteness, the reader may assume that the set of messages is a set of strings, i.e., a free monoid with concatenation over a set of articulatory feature structures. Exponence relations may furthermore be subject to a context restriction. A context restriction *r* is derived by replacing from a concept *r*′ one node or subtree with the symbol “
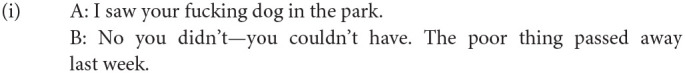
”.^4^ We write *c*→*e*∣*r* to indicate *c* can be exponed by *e* if *r* is satisfied. We understand that an occurrence of *c* as a part of structure *s* satisfies *r* iff. there is a node *c*′ of *s* containing the occurrence of *c* such that replacing that occurrence of *c* with 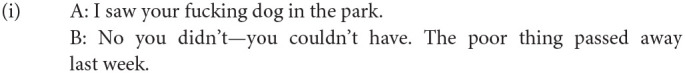 renders *c*′ and *r* identical. For example, the present tense, present, can be exponed in English either with “do” or, if a direct sister to verb phrase, with the null morpheme. The English grammar is captured by the two exponence relations in (2).^5^


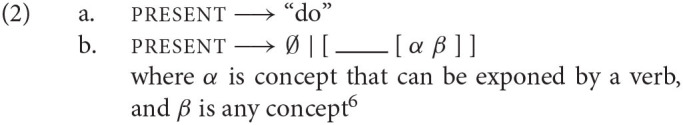


We define a general exponence relation ⇒ that derives sentences by recursive reference to → -relations and a linearization function ℓ (see below). We define an auxiliary notion of $\overset{c}{\Rightarrow }$ where *c* is the structure in which context restrictions apply, and then *c*⇒*s*, (to be pronounced as “*the thought* *c* *can be exponed by* *s*”), is defined as $c\overset{c}{\Rightarrow }s$. The latter, we define recursively as $c\overset{c^{\prime }}{\Rightarrow }m$ iff. either *c*→*m*∣*r* in *c*′ or *c* is structure with the two subconstituent *c*_1_ and *c*_2_ and *m* is the concatenation of *m*_1_ and *m*_2_ in the order ℓ(*c*_1_, *m*_1_, *c*_2_, *m*_2_) of the linearization function^7^ and $c_{1}\overset{c}{\Rightarrow }m_{1}$ and $c_{2}\overset{c}{\Rightarrow }m_{2}$. The set of expressible thoughts is {*c*∣∃*s c*⇒*s*}. Assuming for simplicity that we, like, and linguistics are primitive concepts and exponed by “we,” “like,” and “linguistics,” respectively, (3-a) is predicted to have two exponents, (3-b) and (3-c).


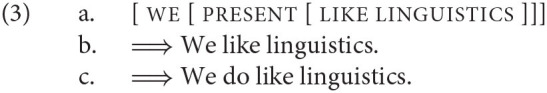


Example (3) illustrates a problem: (3-b) and (3-c) intuitively do not mean the same. We still need to account for the fact that only (3-b) may be used to express (3-a). We do so by introducing the notion of *compression* to capture manner implicatures (Rett, 2014, and others). We define a cost function as a *k* that maps a message *m* to its cost k(*m*) with k(∅) = 0 and k(*ab*)≥k(*a*) and k(*ab*)≥b. Furthermore we assume that there is a measure of probability of understanding specific thoughts by the receiver of *m*, i.e., a probability distribution *P*(—∣*m*) on the set of expressible thoughts.

Finally we define a *compression function* as follows: E is a compression function iff. E maps any expressible thought *c* to an exponent E(*c*) [i.e., *c*⇒E(*c*)] and there is no cheaper message with higher likelihood of reconstructing *c*:^8^ i.e., there must be no *m* with *c*⇒*m* with *k*(*m*) ≤ *k*[E(*c*)] and *P*({*c*}∣*m*)≥*P*[{*c*}∣E(*c*)]. Intuitively, a compression function can be understood as a licit way of relating thoughts to exponents. We will say that *c* can be *expressed* as sentence *s* if there is a compression function mapping *c* to *s*.

Compression can derive the manner implicature in example (1) as follows: If (3-a) is the only expressible thought, there are two candidates for a compression function: E_1_ maps (3-a) to (3-b), and E_2_ maps (3-a) to (3-c). Assume also that both (3-b) and (3-c) have probability 1 of being understood as (3-a), as seems reasonable if (3-a) is the only thought that could be expressed as either. But if the cost *k*(“do”) is greater than 0, the cost of (3-b) will be less than the cost of (3-c). These assumptions taken together entail that E_2_ is not a compression function because the cost of (3-c) is greater than that of (3-b) while the likelihood of *c* being understood is the same for both sentences. E_1_, however, is a compression function.

If we assume that any pronounced material has some cost, we predict that ellipsis must be obligatory whenever it can be reconstructed.^9^ Specifically, we predict that generally periphrasis cannot express a thought *c* if there is a compressed way of expressing *c*, even if periphrasis is a possible exponent of *c*. Two classical cases that can be handled in the same way are the relation of “kill” to “cause to die” (Fodor, 1970), and comparison with antonyms as in “less tall” vs. “smaller” (Bierwisch, 1967; Rett, 2014; Moracchini, 2019, and others). To derive that periphrastic forms like (3-c) are not ungrammatical, but have a marked meaning, we adopt the idea of Rett (2014) that periphrasis and other apparent optional pronounciation indicates the presence of additional structure.^10^ Specifically, we predict that the thought (5) could be exponed as (4-c), but not as (4-b) because the context restriction of relation 6 isn't satisfied.


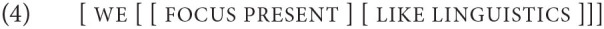


Across languages we expect compression and especially non-pronounciation to vary depending on the morphology of a language. But does tenselessness in languages like St'át'imcets (Matthewson, 2006), Halkomelem and Blackfoot (Ritter and Wiltschko, 2009) really only amount to the non-pronounciation of tense? Before we return to tense further, consider briefly the subject of a verbal predicate, i.e., *subjectlessness*. A rich linguistic literature shows that universally subjects are present,^11^ but they may be unpronounced in some languages and environments. Such *null subjects* are indicated as *pro* or *PRO* following (Chomsky, 1981). These findings corroborate the Meaning First approach because it assumes that at the conceptual level the subject position must be instantiated independently of whether its content is pronounced. Furthermore work on *pro-drop* languages finds a relationship between morphological properties and the pronounciation of the subject as the Meaning First approach predicts (Rizzi, 1982; Alexiadou and Anagnostopoulou, 1998, and others). Now let us return to tenseless languages where there is currently a debate: On the one hand, Matthewson (2006) and others present evidence that tenselessness is similarly superficial as subjectlessness is. On the other hand, Ritter and Wiltschko (2009) and Wiltschko (2014) argue that tense is only a subcategory of an *event anchor* along with person and location, and that at least one subcategory of the event anchor must be pronounced. The Meaning First approach is compatible with various outcomes of further empirical investigation. For example, we could assume that a specification of an event anchor triplet is universally present at the conceptual level, but that the values of the time, location and person specification are predictable from one-another.^12^ Compression would then predict that only one of the three specifications should ever be expressed (and none in the environments where infinitives occur).

Nominal conjunction provides a case where generally compression is not optional. Consider uses of *and* combining two proper names as in (6-a), where Boolean *and* is not directly applicable. Adapting a proposal by Winter (1996) and Mitrović and Sauerland (2016) argue that (6-a) should be analyzed as sketched in (6-b), where the subset relation applies to yield two constituents that Boolean *and* can apply to.^13^


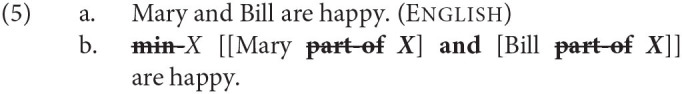


The part-of relation can be expressed by *of* in other environments in English, and the closely related subset-of relation can be expressed by *every* and other universal quantifiers according to generalized quantifier theory (Barwise and Cooper, 1981), but not in (6-a). Mitrović and Sauerland (2016) argue that Japanese and other languages contrast with English with respect to which elements of a nominal coordination are expressed. For example, nominal coordination in (7-a) contains two occurrences of *mo* which in other environments can also express universal quantification. Hence, Mitrović and Sauerland (2016) provide the analysis sketched in (7-b) where **mo** expresses the subset relation while the Boolean conjunction remains unexpressed.


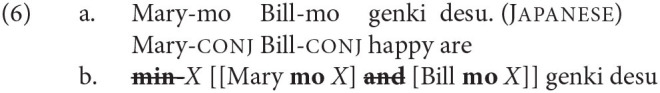


That compression applies with different results in different languages is, we believe, frequently the case. The cross-linguistic variation of kind reference is another case in point. Chierchia (1998) discusses contrasts such as (8) between English and Italian. He argues that while in Italian the definite determiner must be pronounced, kind reference in English also involves a definite determiner, but one that is not pronounced. If Chierchia's analysis is correct, pronounciation of the definite marker as *the* is blocked in English (8-a), but required in Italian (8-b).


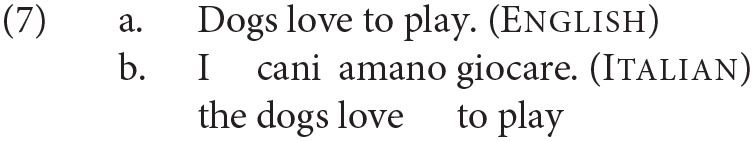


Beyond the concrete analyses discussed in this section already, compression also has some theoretical utility for understanding the *Effability Hypotheses* that each conceivable thought can be expressed verbally (Katz, 1976; von Fintel and Matthewson, 2008, and others). Our formal notions of thought and compression allow us to distinguish three different flavors of effability. Two flavors of effability arise directly from the Meaning First approach: *Conceptual Effability* says that for any two possible worlds *w*_1_ and *w*_2_ that are perceptually discriminable there is a thought *c* that is true in *w*_1_ and false in *w*_2_ or vice versa. *Compression Effability* is satisfied if there exists a compression function for the set of all thoughts. Our model does not predict either flavor of effability to be necessarily true. But furthermore, even if a language satisfies both conceptual and compression effability, there is a third sense of effability that it may not satisfy. Namely with *Transmission Effability*, we mean that any thought *c* can be communicated by the message E(*c*)^14^. For example, the compression function that maps any thought to silence, ∅, satisfies compression effability, but cannot satisfy transmission effability unless there is only a single thought. In sum, the Meaning First approach allows more specific flavors of effability, but to what extent the different flavors of effability are satisfied remains an empirical question.

The presence of unpronounced material in linguistic structure, and in particular in the three cases of compression presented above, is debated in linguistics. The debate concerns the relation between conceptual structure and articulated form: is the relation between the two rather “simple” and “direct,” or rather is the thought internal concept composition uniform? Much research in linguistics has favored a one-to-one mapping between the primitive concepts and articulated linguistic elements as illustrated by (7) –for example, both Cognitive Grammar (Langacker, 1987) and Montague Grammar (Montague, 1970) place great value on such directness. From the Meaning First perspective, though, the motivation for a one-to-one mapping seems questionable: on this view, the one-to-one mapping would be stated as a requirement that each primitive element of a thought must be articulated if a thought is communicated^15^. But while communication is subject to the optimization principles of information theory (Shannon, 1948; Hale, 2016), we would be surprised to find that the thought system is subject to information-theoretic principles in the same way. Engineered solutions frequently use different data formats for internal processing and for transmission to another machine; one highly redundant representation that can be easily processed, the other highly compressed. Our approach places much higher value on the uniformity of operations at the conceptual level since it recognizes as separate the compression system language and the underlying thought structures. Compression distinguishes the Meaning First approach from other approaches to grammar including Generative Semantics because compression allows the different possible exponents of a structure to receive different interpretation, which the model of Katz and Postal (1964) excluded. At this point, evidence for compression remains preliminary since thought structures need to be ascertained.^16^ We put compression aside for now, and focus in the remainder of the paper on the three other sources of evidence of the Meaning First approach mentioned in the introduction.

## 3. Scope Relations

**Figure 2:**
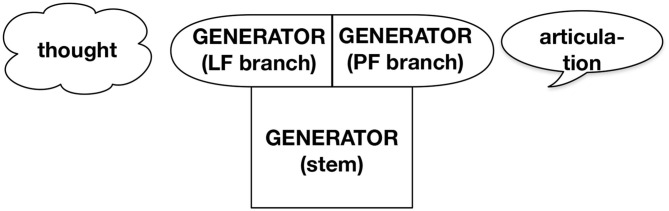
The T-model of language assumes that structural representations are generated within language and produce a thought-articulation pair.

For about 50 years the *T-Model*^17^ architecture of grammar sketched in Figure 2 has held sway: structures are generated within grammar itself and then are fed to two interfaces, the LF-interface with the conceptual system and the PF-interface with the systems for articulation and perception.^18^ The Meaning First approach instead advocates an architecture where a conceptual representation is generated outside of grammar, which the linguistic system then packages for communication. In this section, we address the empirical argument in favor of the T-model based on scope relations (Chomsky, 1970; Jackendoff, 1972). Chomsky (1970) actually discusses two arguments in favor of the conclusion that grammatical operations affect interpretation, but the second one based on intonational focus has already lost its force. Specifically the discovery that focus can be articulated solely by segmental affixes in, for example, Wolof (Rialland and Robert, 2001) and Chickasaw (Gordon, 2008) has supported an analysis of focus in English as affixes too, but ones that are articulated by intonational means. The affixal analysis of focus does not require the T-model because an affix can uncontroversially expone a part of a conceptual structure.

Consider now the argument from scope relations in favor of the T-model. It is based on the empirical generalization that the linear order of exponents (also called *overt word order*) affects scopal relations. Initial empirical evidence came from data such as (9) (Jackendoff, 1969; Chomsky, 1970), where the word order of the quantifier *many* and negation correlates with the preferred interpretation.^19^


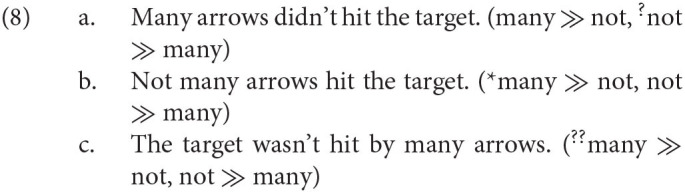


Paradigm (9) argues against the Generative Semantics account where (9-b) and (9-c) articulate the same conceptual structure as (9-a). But the Meaning First approach assumes that three different conceptual structures underlie the sentences in (9) (Sauerland, 2018): In (9-b), the position of *not* would reflect different positions of negation in the conceptual structure, and the passive morphology in (9-c) indicates the presence of a primitive concept, pass (Alexiadou et al., 2015 and others), not present in (9-a).

Though most work on the syntax-semantics interface has assumed the T-model, only few of the results accomplished actually depend on this architecture as far as we know. We are optimistic that it is possible to reconsider such results fruitfully within the Meaning First approach. Specifically one influential paradigm of data supporting the T-model was presented by Fox (2000). Fox's analysis takes the T-model for granted, but is interesting because it crucially relies on the distinction between overt and covert operations that is available only in the T-model (see also Reinhart, 2006). But Sauerland (2018) suggests an approach to Fox's data within the Meaning First approach that might hold some advantages over Fox's account. In sum, we have shown that the two main existing arguments from scope in favor of the T-model are actually consistent with the Meaning First approach, and points to avenues for further empirical investigation.

## 4. Late Insertion and the Division of Content

In this section, we argue that the Late Insertion of lexical material the Meaning First approach allows a natural account of the division of content between logical and socio-emotive aspects. We will contextualize our proposal in light of discussions within Distributed Morphology on *Late Insertion*, but note that the work of Bock and Levelt (1994) and others on speech production bears many similarities to Distributed Morphology. Distributed Morphology is a realizational theory of morphology. It assumes that there is a separation between the syntactic-semantic content of morphemes and the articulatory instructions required for its exponence for communication. *Vocabulary Insertion* describes the provision of phonological exponence to morphemes. For example, vocabulary insertion realizes the syntactic and semantic features for 3rd person singular in combination with present tense information with verbs like *see* in English by realizing them with a voiced “*z”*. The term *Late Insertion* indicates that vocabulary insertion takes place after a structural representation is formed. Within Distributed Morphology it is debated whether Late Insertion applies universally to all morphemes including roots. Unlike Distributed Morphology, the Meaning First approach is committed to *Universal Late Insertion* because the Meaning First approach locates the structure generation outside of grammar while Distributed Morphology views it as part of grammar. We present existing evidence and argue that the Meaning First approach overcomes a conceptual problem that *Late Insertion* poses for Distributed Morphology. Then we develop a new argument for *Universal Late Insertion* from the division of content.

While Late Insertion of functional and inflectional morphemes is a hallmark of Distributed Morphology, Late Insertion of roots has been controversial. Marantz (1995) argued that the process of Late Insertion applies both to inflectional and other functional morphemes as well as to roots. The Late Root Hypothesis was substantiated on the basis of a series of arguments. First, Marantz argued, phonological material seems irrelevant for the syntactic derivation. Second, meaning differences between roots are not relevant for syntax (e.g., **cat** vs. **dog**). Thirdly, while the difference between roots and functional morphemes is descriptively useful, there is little evidence to argue that it affects the basic generative system. These arguments led Marantz to propose that Vocabulary “*is the output of a grammatical derivation, not the input to the computational system”* (Marantz, 1995, p. 411) and thus universal late insertion. Marantz (1995) notes that universal late insertion in Distributed Morphology requires a link between phonological and semantic information that is not mediated by the syntax:^20^ two concepts that have identical syntactic behaviors, e.g., **cat** and **dog**, are represented in the same way in the syntax, but have different pronounciations and interpretations. Marantz (1995) leaves the *cat-dog problem* open while Harley (2014) suggests that the root terminal nodes could be notated via indexes. Universal late insertion was criticized and rejected in later work by Embick (2000) and Embick and Noyer (2007), who adopt late insertion for functional morphemes only. But more recently, Pfau (2000), Haugen and Siddiqi (2013), de Belder and van Craenenbroeck (2015) and others argue in favor of universal late insertion. The Meaning First architecture requires universal late insertion after formation of a structure, but it avoids the cat-dog problem of Marantz (1995). The realization process maps semantic concepts to their articulation instructions, e.g., **cat** → “*cat, ”* while **dog** → “*dog”*.

The Meaning First approach also sheds new light on the possible identification of *grammatical* features. It has been observed that the distinction between innate concepts vs. those that are based on experience (Carey, 2009, and others) predicts quite well which features grammar can access; for example, the innate conceptual distinctions between a single object and a plurality or between the present and the past frequently are accessed by grammar, while the distinction between *cat* and *dog* and others like it never are.^21^ Most approaches of Minimalist syntax assume that there is an innate finite inventory of grammatical features (Chomsky, 2015) (though e.g., Biberauer and Roberts, 2016 disagree). As we mentioned already, Marantz (1995) implements the sole sensitivity of syntax to grammatical feature by universal late insertion. But on the Meaning First approach assumes any information in a concept could be available to grammar. If the link between grammaticality and innateness of a feature was incorrect (Biberauer and Roberts, 2016), this would therefore speak in favor of the Meaning First approach. However, we also think that if the link between grammaticality and innateness of a feature is correct, it can be insightfully implemented within the Meaning First approach. Specifically, being a grammatical feature in the terminology of section 2 above, means that a conceptual property can occur in the environment condition of exponence relations. Chierchia (2013) and Del Pinal (2019) argue that the innate features are also logical and not affected by contextual adjustment. Therefore, we suggest that environment restrictions in the Meaning First approach are restricted to logical concepts to capture the syntactic status of grammatical features.

We now argue that the Meaning First approach can provide an account of the division of semantic content between logical and socio-emotive meaning widely assumed in socio-linguistics (Eckert, 2018). The account is based on the assumption that the signals language relies on for communication (i.e., the exponents) can in addition to their exponence relation associate with socio-emotive impact. We assume the socio-emotive associations are direct, non-compositional links between an exponent and socio-emotive similar to associations from facial expression or clothing style serve to communicate socio-emotive aspects. We show that a model of socio-emotive content within the Meaning First approach based on the assumption of exponent-based association predicts some key properties dividing logical and socio-emotive content.

Consider first that any use of the morpheme *dog* carries with it further meanings: it may be realized with a gesture (*dog* + smile), with a phonetic variant (Labov, 1966), with an emoticon *dog :-)*, quoted “*dog*,” spelled *d-o-g*, or lengthened *dooog*, and all variants serve to communicate some further layers of meaning such as the speaker's attitude toward dogs or a particular dog, but also other aspects of the speaker's state of mind. Semantic research has found, though, that semantic meaning can be broadly divided into two; we use the terms logical and socio-emotive meaning (Potts, 2005; Gutzmann, 2015; Eckert, 2018).^22^ We assume that the thought structure constitutes the logical meaning, while the socio-emotive aspects are arise from the use of an expression.^23^ A useful criterion to distinguish between the components is provided by structures that require an identity of logical meaning (Potts et al., 2009). For example, socio-emotive meaning can be added to the second occurrence of *president* in (10), but not logical meaning.^24^


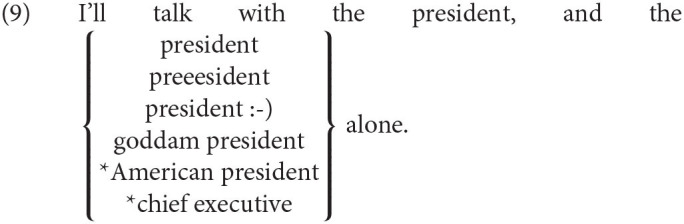


The Meaning First approach can predict the relevant identity requirement by assuming the conceptual representation sketched in (11) for all exponents in (10), where the concept **president** occurs in two positions. We propose furthermore that socio-emotive content can affect the process of realization at each occurrence.


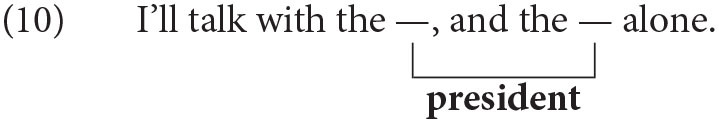


Our novel evidence in (12-a) shows that this mechanism can also lead to clear-cut cases of late insertion of a root.^25^ In (13), ellipsis applies in the question by B, but the pejorative component of *Köter* (“fleabag”) is not present in B's utterance.


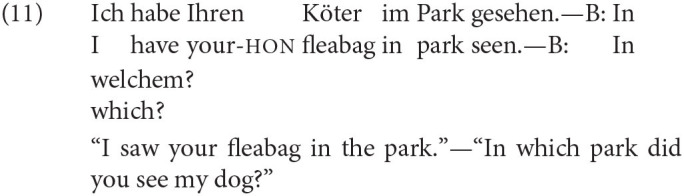


Adopting ideas of Adger and Smith (2010), we assume that German provides at least two different exponents for the **dog** concept. We write these as in (14), where in (14-a) socio-emotive side effect of the exponent are indicated as explained in the following. For ellipsis licensing, we assume that only the conceptual level matters.


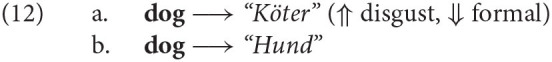


We use the label *Intrusion* for cases where socio-emotive content affects exponence. Consider briefly a starting point toward a model of intrusion: Assume that there is a function *a* mapping an exponent *e* and a socio-emotive property *p* to one of −1, 0, and 1. The arrow notations in (14-a), we take to indicate that *a*(“*Köter”*, disgust) = 1 and *a*(“*Köter”*, formal) = −1, while the value of *a* is 0 whenever it is not indicated. For some properties, speakers aim for the closest possible match between concept exponed and the socio-emotive properties of the exponent. This is responsible for intrusive use of *Köter* instead of *Hund* for **dog** by individuals disgusted by dogs. Furthermore speakers can adopt targets for properties like “formal” that they consider appropriate for a specific situation, e.g., τ_formal_ = 1. If we evaluate closest fit by the Euclidean distance between the target disgust-formal pair and the disgust-formal pair of the exponent, we correctly predict intrusive uses of “Hund” by dog-haters in situations they perceive to require formal speech. The model we layed out is evidently a toy model that is insufficient to capture all relevant phenomena (see Gutzmann, 2019 and others for more sophisticated models, but starting from different assumptions). But we find the restritiveness of the toy-model attractive, in particular it predicts that socio-emotive meaning can be restricted to only the current exponent and the concept exponed. If this restriction is corroborated by more data, socio-emotive intrusion may represent a residue of prelinguistic communication abilities.

Via intrusion, the Meaning First approach allows contentful late insertion of socio-emotive components of meaning, and there is empirical evidence for it. The Meaning First approach also predicts, as we saw, crucial aspects of socio-emotive meaning components,^26^ namely their non-compositional nature and their lack of interaction with other semantic content of the sentence.^27^

## 5. Multiple Languages

In this section, we will focus on the third prediction of the Meaning First approach, namely that thought can simultaneously access two language systems.^28^ We rely on recent work on multilingual individuals that has shown that such individuals frequently mix two or more languages, but also that such mixing is subject to non-trivial structural restrictions (López et al., 2017 and others). The evidence supports a view where some aspects of grammar are language independent and therefore also apply when two languages are mixed. Previous theoretical work on multilingualism has focused on Distributed Morphology, which we discussed already in the previous section. Our view in this section is that much of the evidence is compatible with both Distributed Morphology and the Meaning First approach, but some more recent evidence from code-blending specifically supports the Meaning First approach. We first briefly discuss code-mixing and then focus on code-blending.

Work on code-mixing has shown that bilinguals use different vocabulary items to realize a unified abstract structure. For example, Alexiadou (2017) and Alexiadou and Lohndal (2018) show that bilinguals create novel forms consisting of roots from one language combined with affixes from another. The grammar of such forms is governed by rules, e.g., Alexiadou and Lohndal (2018) discuss grammatical gender in Greek-German code-mixing: a stem like *Kass* (“cash register”) from the feminine class in German must be marked with feminine morphology *i Káss-a* (“the cash register”) in code-mixing, even though the Greek translation *tami-o* (“cash register”) belongs to the neuter class. Cases of such sub-lexical mixing argue that language mixing is based on an integrated language-independent structural representation as both Distributed Morphology and the Meaning First approach assume. The realization mechanism can then be switched at certain points while a structure is realized (López et al., 2017).

Code-blending provides evidence that favors the Meaning First approach over Distributed Morphology. Code-blending involves a mix of signed and spoken utterances which are to some extent produced simultaneously in the two modalities by bimodal speakers (Emmorey et al., 2008, and others). Branchini and Donati (2016) report examples like (15) where the word order of negation and verb differs in the two simultaneous utterances, namely the correct word order of the individual languages is used for both.


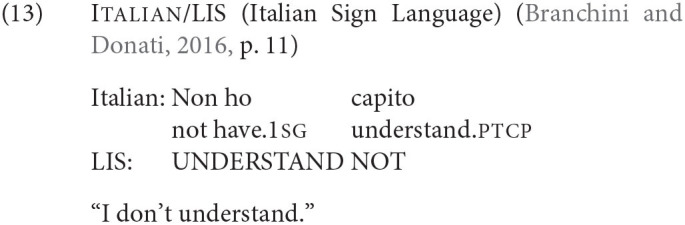


**Figure 3:**
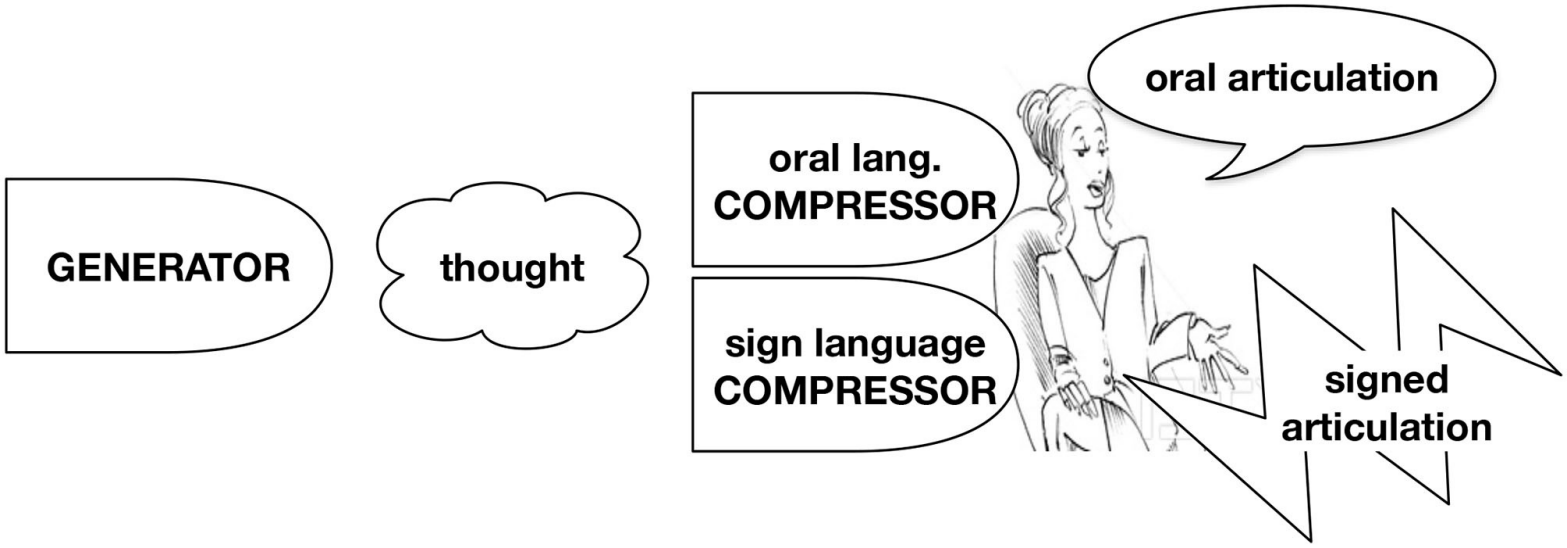
The Meaning First model of bimodal speech: Two compressors realize a single thought representation simultaneously in the two modalities.

Though Branchini and Donati (2016) suggest an analysis of their data within Distributed Morphology, this would require an extension of morphology to word order variation. As Figure 3 illustrates, the Meaning First approach, however, predicts data like (15) if two articulation mechanisms operate in parallel and each produces the appropriate word order.^29^

The view of Branchini and Donati (2016) would furthermore lead to two distinct logical form representations for the LIS and Italian sentences, which could have different interpretations. But the Meaning First approach predicts that the interpretations must be parallel since both articulations derive from the same conceptual representation. The latter view in addition to being more plausible, is empirically supported by some still unpublished work: (Lillo-Martin, 2019, slide 39) reports data from idiom interpretation in bimodals. Namely, she tested English-ASL bilinguals on utterances of an ASL sentence simultaneous with an English sentence that contains an idiom. For example, she reports that a simultaneous utterance of ASL *NOT WORRY SMALL PROBLEM* with English *Don't cry over over spilled milk*.—i.e., meaning parallelism without morphological parallelism—is judged much more acceptable than the reverse. The Distributed Morphology view does not predict an interpretive link of this kind: the English grammar should independently of the ASL grammar select either the idiomatic or non-idiomatic interpretation. However, the Meaning First approach predicts that the interpretations must be the same since both structures must derive from the same thought structure.

## 6. Conclusion

We have presented three arguments for the Meaning First approach to grammar. This approach proposes that language structure derives from the structure of logical thought as sketched in Figure 1. But not all pieces of a thought structure need to be realized in language: Many pieces may be predictable from the presence of some key fragments with enough reliability to allow communication to be successful. Therefore, realization in the Meaning First approach consists primarily of compression. At the same time, other cognitive faculties may intrude on the realization of thought structures in language. Especially, we have talked about socio-emotive attitudes, which often find expression in or alongside language, but interact only in limited ways with logical meaning, if at all. We conclude that a Meaning First approach to grammar should be considered in more detail in the future as we plan to do.

A central goal of linguistic theory is to predict the set of possible languages; i.e., those learnable by typical individuals. How does the Meaning First approach constrain the set of possible languages? In two important domains, it can build on existing lines of research: the work by Gärdenfors (2000) on constraints on the set of primitive concepts and the work in syntax and Distributed Morphology on linearization and realization relations (e.g., Embick, 2010; Richards, 2010; Smith et al., 2019, and others). The former govern how much information can be packaged into a single concept, while the latter govern to what extent this packaging can be altered by compression. As an example of this interaction between the conceptual and morphological constraints, consider the results of Bobaljik (2012) on adjective gradation. Bobaljik argues that the superlative cannot be a primitive concept, but is always conceptually represented as a complex of the comparative concept and maximality as in (16-c). He furthermore argues that the morphology is universally constrained in such a way that a exponence rule for an adjective concept cannot have an environment condition that is only satisfied by the positive and the superlative conceptual structure, excluding the comparative. With these assumptions, Bobaljik derives the *ABA universal he establishes, namely that the hypothetical ABA-language in (16) with the gradation sequence bonus–melior-*bonimus is not a possible human language.


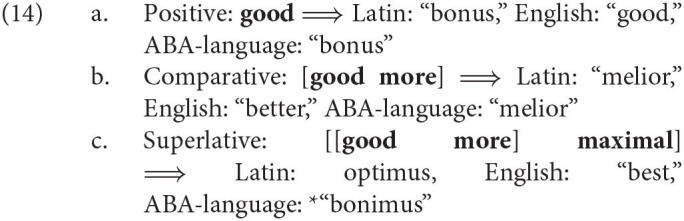


Painstaking empirical work such as Bobaljik's remains necessary to establish constraints on how both basic concepts and the compression component are constrained on the Meaning First approach as much as for other approaches. These remarks apply *mutatis mutandis* to constraints in other components of the model such as concept combination, intrusion, and the possible cyclic conception of the Meaning First approach. We expect though that the focus on primitive concepts and their exponence on the Meaning First approach provides a better space to integrate such work than frameworks like Montague grammar (Montague, 1970) that do not allow for a morphological component.

The Meaning First approach therefore has major repercussions for how we investigate language and thought. First of all, we cannot separate the study of the two if grammatical structure is derived from thought structure. But the presence of compression and intrusion also means that a thought structure may differ substantially from the sentence used to communicate it. In particular, the thought structure may be much more complex—for all we know, language may achieve compression rates of 10 primitive concepts to one morpheme or even 100:1. Furthermore, the Meaning First approach provides two different avenues language may affect thought (i.e., the phenomenon of *linguistic relativity*, Deutscher, 2011): On the one hand, languages may express different logical or acquired concepts and therefore require speakers of one language to attend to a specific distinction more frequently than speakers of other languages, and thereby become more practiced in drawing that distinction. On the other hand, compression to language itself may support thought as a kind of mnemonic. Specifically we speculate that complex thought representations may be difficult to process, but their processing aided by temporarily storing parts of them by means of a compressed articulatory representation. For example, inner speech aiding memory may explain the effect of language on certain theory of mind tasks (de Villiers and de Villiers, 2000; Sauerland, 2020, and others). Most importantly, though, the Meaning First approach brings to attention that we know very little about the principles of thought structure and the process of compression. The potential examples of compression we discussed in the section 2 and Bobaljik's case illustrate some of the analytical techniques to make concrete progress by comparing different languages and seeking overall simplicity. We think exciting progress can be made by applying such techniques widely as well as by indentifying further sources of evidence.

## Data Availability Statement

The original contributions presented in the study are included in the article/supplementary material, further inquiries can be directed to the corresponding author/s.

## Author Contributions

US and AA conceptualized the paper and wrote the final paper. US wrote the first draft of the paper. Both authors contributed to the article and approved the submitted version.

## Conflict of Interest

The authors declare that the research was conducted in the absence of any commercial or financial relationships that could be construed as a potential conflict of interest.

## Footnotes

^1^Our view on the thought-language relation is related, but distinct from the view that language itself is an instrument of thought of Chomsky (2015) and others. Everaert et al. (2015) argue that on Chomsky's view language itself has a property (namely, hierarchical structure) not well-suited for communication. On our view, hierarchical structure is a property of the thought system, but otherwise we agree with Chomsky's view.

^2^The Meaning First approach requires that restrictions on possible concept combination must be independent of language. But the independence is difficult to verify since most work on concept combination that we know of is based on the formal semantics of language and in formal models the borderline between properties of language and of the conceptual calculus is difficult to pin down. For example, type-theoretic restrictions can be captured in the formal ontology as well as in a categorial grammar (Ajdukiewicz, 1935, and others).

^3^In algebra, our conception is called the *minimal commutative free magma* over *C*. The commutative magma differs only for “self-merge” of a concept *c* with itself from Chomsky's Merge (Sauerland and Paul, 2017). We prefer the algebraic conception for its greater clarity.

^4^Evidently, “” must not be a primitive concept to avoid mix-ups.

^5^Our treatment of exponence is abbreviated, which forces us to state the contextual restriction in 6 in an inelegant statement referring to possible exponents of α. See especially the contributions in Trommer (2012) for a discussion of how exponence of one concept can be linked to the exponence of others in the same structure.

^7^The concept of linearization would need to be expanded for phenomena in language that are not linearly ordered. But we think such an extension would be straightforward and omit it here for perspicuity.

^8^Note that compression derives much of the effect of morphological specificity (Bobaljik and Sauerland, 2018 and others): If two possible exponents have the same cost, the more specific one must be used. We hope future work will compare this corollary with standard specifity empirically. In future work, we also plan to consider a version of compression that is sensitive not to the likelihood of reconstruction of *c*, but to the expected utility of the reconstructed thought(s).

^9^The same applies to non-pronounciation in cases not usually referred to as *ellipsis*. One reviewer brings up the interesting case of the English complementizer *that* as a potential counterexample presenting free optionality between pronounciation and non-pronounciation. Levy and Jaeger (2007) show, however, that the presence/absence of *that* is affected by meaning disambiguation. Though it remains to be seen whether this result can carry over to other structures, the evidence we are aware of is in our favor.

^10^The presence of additional structure may also underlie additional positions for temporal, modal, or event information. At least the non-substitutability of *cause to die* by *kill* in (4) and maybe some other classical cases can be accounted for in this way. At this point, we are not aware of any acute problems with treating all cases of non-equivalence of possible exponents as manner implicatures. Finding cases that cannot be accounted for by manner implicature would be very interesting since it might indicate that a primitive and composed concept could be equivalent. (i) John caused Bill to die on Sunday by stabbing him on Sunday (Fodor, 1970)

^11^For ease of presentation, we put aside some cases of infinitivals that are generally taken to have no subject position (Wurmbrand, 2001 and others). These exceptions are fully consistent with the general picture we draw, and strengthen the argument that in most environments silent arguments are represented at the conceptual level.

^12^Predictability applies only at the specified level of granularity, not for the exact time, location, and participants of an event. We specifically assume ±past, ±distal, and ±local following (Ritter and Wiltschko, 2009). It seems plausible to us that relevant past events also are also more likely than present events to have occurred in distal locations and have involved non-local participants, but as far as we know this remains to be empirically studied.

^13^We use sans-serif boldface for logical concepts and strike-out to indicate that a concept remains unpronounced. We tacitly assume here that “min” is the minimum operator and the model-theoretic interpretation of structure (6-b) amounts to the paraphrase: The minimal set *X* that has both Mary and Bill as elements has the property “happy.” See Mitrović and Sauerland (2016) for the details of the proposal that we have to omit here, and Haslinger et al. (2019) for further discussion.

^14^More specifically, *P*[{*c*}∣E(*c*)]>*P*[{*c*′}∣E(*c*)] for any two thoughts *c, c*′. We see this characterization only as a starting point, and future versions could make reference not to the formal identity of *c* and *c*′, but to equivalence.

^15^Within Distributed Morphology, Siddiqi (2006) implements similar intuitions. Benz's (2012) game-theoretic *Error Model* provides a related rationale for the non-articulation of conceptually present material.

^16^There is substantial evidence for compression at the word-level since the work of Zipf (1932). This is not directly related to our concerns, but also corroborates the Meaning First approach.

^17^The term *T-model* is due to Chomsky and Lasnik (1977).

^18^We put aside the addition of the cycle to this conception where the T-model architecture applies within phases (Chomsky, 2015) since our discussion in the following relates to phenomena taking place within a single phase. Also Bobaljik (1995) and Bobaljik and Wurmbrand (2012) propose a departure from the T-model, but still view structure generation as part of syntax unlike the Meaning First approach.

^19^The relevant data become sharper with comparative quantifiers like *more than three arrows* (Takahashi, 2006; Fleisher, 2013).

^20^The assumption that syntax links phonology and semantics is part of the *T-model* of grammar. See also section 3 above.

^21^Adger (2019) discusses the interesting case of symmetry. Why does the difference between symmetric things like faces and asymmetric ones like constellations never enter grammar? We suggest that the notion of symmetry unlike those of object and time is not an innate constituent of our cognitive system, but that our perceptual systems make symmetry universally salient to us. Hence symmetry would be innate, but not in the sense relevant to grammar.

^22^Gutzmann (2015) uses the terms *truth-conditional* for our *logical* and *use-conditional* for our *socio-emotive*. Two criteria distinguishing the two types is that socio-emotive content generally doesn't compositionally interact with other parts of the sentence and that it is irrelevant to many semantic identity requirements as we discuss in the following. Both tests are as far as we know not unequivocal and can be difficult to apply. Further criteria such as translatability (McCready, 2014) may also exist.

^23^Our view might allow us to capture medical conditions such as copralalia as a dissociation of the two logical and socio-emotive systems (Van Lancker and Cummings, 1999 and others).

^24^These data are due to Potts et al. (2009) except for the second and third variant, which are based on Tieu et al. (2017).

^25^The English example (12) from Potts et al. (2009) is related, but German packages *fucking dog* into the single noun *Köter* (“cur/fleabag”) (Gutzmann, 2011). While the gloss *cur* suggests mixed race, the German *Köter* does not carry such a implication, but may be related to *Kot* (“excrement”/“dirt”) or the barking sound.

^26^A reviewer raises the possibility of adding a social meaning system to the phonology branch within a T-model grammar. We think it would be interesting to spell out such a system, but expect that once spelled out such a model would bear key similarities to the Meaning First model. Specifically it would need to make sure that a particular use of *Köter* (“cur”) needs to relate to a particular referent.

^27^Furthermore the non-translatability of socio-emotive content (McCready, 2014) is also captured by the link between intrusion and specific exponents in the Meaning First approach.

^28^We believe that other transfer phenomena in bilinguals (Jarvis and Pavlenko, 2008) also are supportive of the Meaning First approach, but less unequivocally so than the evidence we discuss here.

^29^We expect that the independent operation of two articulation mechanisms will be constrained by performance factors (Petroj et al., 2014). Rodrigo et al. (2019) provide corroborating evidence from priming that bilinguals represent sentences independent of word order.
